# Case of a Female, the Mother of Seven Children, Who Never Menstruated

**Published:** 1853-05

**Authors:** S. T. Gregory


*Case of a Female, the mother of seven children, who had never menstruated.*—By S. T. Gregory, M. D. With Remarks by C. D. Meigs, M. D., Professor of Midwifery in Jefferson Medical College, Philadelphia.

To Isaac Hays, M. D.
*Editor of the Am. Jour. Med. Sci. March,* 20 1853.

Sir : I lately received a letter from Dr. Gregory, of Warrenton, Missouri, which appears to me so interesting as to merit a place in your Journal. It does not seem necessary to publish the whole of the communication, with which Dr. Gregory has favored me. I therefore beg to offer for your press the following extracts :—

*Warrenton, Missouri, Jan.* 18, 1853

Prof. Charles D. Meigs, M. D.,

Dear Sir : *Quere.*—"Is a woman who never menstruatad capable of conceiving and bearing children?" This is a question that has oftentimes presented itself to my mind ; and, from all the information I possessed on the physiology of menstruation, I unhesitatingly decided in the negative. And, I will farther state that, for fear my opinions upon the subject were incorrect, or too hastily arrived at. I spared no pains nor labor to consult the highest and most reliable authorities (both French, English and American), but could find but little, however, in favor of the proposition ; and nothing in the main, satisfactory or conclusive. It was my good fortune (if I may be allowed the expression) in the summer of 1846, to meet with a case just in point. I was consulted by

Mr.——, an account of his wife, who he informed me, had been complaining for some weeks of the following symptoms: costiveness, headache, sleeplessness, eructations, &c., &c., Thinking that the symptoms as described, were the results of indigestion, I prescribed a few pills of extract. colocynth. comp. with extract. hyos., and an antacid and tonic, to be taken *pro re nata.* After recommending the above treatment, I accidently asked him the further question, "if his wife was regular in her monthly courses?" and to this he gave me the following reply: "In that respect, Doctor, my wife is very curious. From all I can learn, she is not as other women. She has never been in that state since we were married, and she assures me that she never was before marrying." He stated that she was the mother of six living children (all boys), and that she was then far advanced in pregnancy. As I had attended her in several previous labors, he also requested me to attend her again. To this I readily consented, and in due time, I was called upon. Thinking that he had probably been deceived in relation to the condition of his wife, I thought this would be a most auspicious opportunity to learn the facts from the lady herself. She was safely delivered of a well-formed male child, without an untoward symptom. So soon as delicacy and a sense of prudence would admit, I related to her the conversation I had had with her husband some months previous, and she assured me that all he had said on the matter was true to the word.

She stated that she had never in her life been unwell, like other women. She had never experienced the usual symptoms preceding menstruation. She had never been troubled with any vicarious discharge, except twice, when she threw up some dark grumous blood. Her general health had usually been good—so much so, that she had been able, most of the time, to attend to domestic affairs without much assistance. Now this was certainly a novel case to me, and one that I felt much interest in. It also satisfied me that, while it is almost as natural for a woman to menstruate as it is for her to breathe, yet now and then cases do appear where women who have never menstruated may conceive and bear children. These cases may certainly be considered anomalous.

In conclusion, let me ask you again, to pardon me for thus intruding upon your time and patience with this uninteresting letter; and, with considerations of the greatest regard, believe me yours, very respectfully,

S. T. Gregory.

After reading the above extract from Dr. Gregory's letter, it appears to me necessary only to make the reflection that as fecundation without a previous act of ovulation, is not to be deemed possible, this lady was the subject of the germ-producing act as fully as any other woman can be—and that, as the menstrual discharge is nothing more than a simple physiological effusion of blood, determined by the processes of ovulation, it is for many women indifferent whether the sanguineous discharge does not coincide with the oviposit.

Probably, menstruation consists, more essentially in the periodical oviposition, than in the bleeding from the womb that attests the progress of that important office. In Dr. Gregory's patient, no visible signs of menstrual hyperæmia have ever presented themselves, save only those we observe as to the conceptions and gestations, which are the most characteristic of them all.

In this case, we have no greater reason for surprise at the absence of the catamenia, than we have in the instances, very numerous, of women who never *see* after the first conception, until, after numerous lyings-in, they cease to bear children, whereupon they prove to be exactly regular, as it is called. But such women were always exactly regular in the oviposition—though they never exhibited any *show* of it until they ceased to conceive.

Upon the whole, Dr. Gregory's case is well worthy of perusal. But let the reader beware not to found upon these interesting facts a decision as to the question, whether a non-menstruating young woman is marriageable or not. I have met with too many instances of total absence of the uterus in married women, not to feel how necessary it is, in all such doubtful questions, to have the facts of the case clearly understood, before a professional sanction is given to a union that cannot but produce unhappiness, where abnormal development by default, renders the marriage hopelessly sterile, and the rite impossible.

I am sir, with great respect, &c., &c.,

C. D. MEIGS.

